# miR-99b-5p inhibition drives apoptosis and tumor shrinkage in triple-negative breast cancer: functional characterization through AGO2-RIP-seq and mechanistic insights

**DOI:** 10.3389/fonc.2026.1788447

**Published:** 2026-04-22

**Authors:** Senem Noyan, Kubra Nur Kaplan Ilhan, Muge Ocal Demirtas, Begum Karaoglu Dallı, Bora Ergin, Rabia Sen, Hakan Gurdal, Bala Gur Dedeoglu

**Affiliations:** 1 Biotechnology Institute, Ankara University, Ankara, Türkiye; 2 Medical Biology Department, Yüksek İhtisas University, Ankara, Türkiye; 3 Intergen Genetics and Rare Diseases Diagnosis Center, Ankara, Türkiye; 4 Faculty of Medicine, Department of Pharmacology, Ankara University, Ankara, Türkiye

**Keywords:** AGO2-RIP-seq, apoptosis, breast cancer, miR-99b-5p, TRAIL-R, tumor suppression

## Abstract

**Background:**

Dysregulated microRNAs (miRNAs) are critical contributors to breast cancer biology, yet the functional roles of many remain incompletely understood. miR-99b-5p has been widely characterized as a tumor-suppressive miRNA in numerous cancer types, where its expression is consistently reduced in tumors compared with normal tissues. In contrast, our analyses of breast cancer datasets revealed a unique expression pattern: miR-99b-5p is significantly upregulated in breast tumors, suggesting a context-dependent oncogenic function. In this study, we identified miR-99b-5p as an oncogenic driver in triple-negative breast cancer (TNBC).

**Methods and results:**

TCGA-based expression profiling confirmed its elevated levels in breast tumors. Functional assays demonstrated that downregulation of miR-99b-5p in TNBC cells inhibits proliferation and induces apoptosis, indicating a critical role in sustaining tumor cell survival. To elucidate the molecular mechanisms underlying this activity, we performed AGO2-RNA immunoprecipitation followed by high-throughput sequencing (AGO2-RIP-Seq), enabling unbiased identification of miR-99b-5p-associated transcripts. Pathway enrichment analyses revealed that its direct targets converge on apoptotic regulation, cell-cycle control, and ubiquitin-mediated protein degradation. Mechanistic validation through qRT-PCR, Western blotting, and luciferase assays confirmed that miR-99b-5p modulates the TRAIL-R signaling pathway via DR5 and BAK, attenuating apoptotic signaling. *In vivo* studies using xenograft models established with MDA-MB-231 cells stably expressing miR-99b-5p knockdown showed marked tumor regression, further supporting its oncogenic role.

**Conclusion:**

Collectively, these findings establish miR-99b-5p as a context-specific oncogenic miRNA in breast cancer and a promising therapeutic target, particularly for TNBC, where targeted treatment options remain limited.

## Introduction

Triple-negative breast cancer (TNBC) accounts for 15–20% of all breast cancers worldwide, and it is an aggressive breast cancer subtype with a high incidence of early recurrence. The proliferative and highly metastatic nature of TNBC results in a poorer overall prognosis compared to other breast cancer (BC) subtypes (1, 2). Chemotherapy is the primary treatment approach, as TNBC lacks targets for endocrine and HER2-directed therapies. However, chemotherapy resistance and subsequent disease progression pose a significant therapeutic obstacle, contributing to poor survival and disease relapse (3). There are ongoing efforts to identify actionable targets that take heterogeneity of TNBC into consideration (4, 5). Effective treatments are expected to be the result of understanding mechanistic insights that govern survival, drug resistance, and cell death in TNBC.

MicroRNAs (miRNAs) are short endogenous noncoding RNAs (~20–25 nt) that regulate gene expression primarily at the post-transcriptional level by inducing mRNA degradation or translational repression. Through this regulatory capacity, miRNAs critically influence key cellular processes, including apoptosis, cell-cycle control, and metastasis. Increasing evidence highlights their involvement in the regulation of apoptotic signaling pathways in cancer (6, 7). In line with these observations, our results demonstrate that miR-99b-5p modulates TRAIL-induced apoptotic responses in triple-negative breast cancer (TNBC).

Apoptosis is a natural cell death process that serves as a filter for the biological system to eliminate abnormal cells. Extrinsic apoptosis pathway, which is one of the controlled cell death processes is initiated by tumor necrosis factor–related apoptosis-inducing ligand (TRAIL) and DR4 (TRAIL-R1) or DR5 (TRAIL-R2) (8). The binding of TRAIL trimers to DR4 or DR5 causes receptor trimerization and assembly of the death-inducing signaling complex (DISC). This is followed by FADD-mediated recruitment and activation of caspase 8 and caspase 10. Active caspase-8 and/or 10 then activate executioner caspases such as caspase-3 and initiate the apoptotic cascade (9, 10).

Although apoptotic pathways and miRNA-mediated gene regulation have each been studied to an extent, the miRNA-driven regulation of death receptor– and mitochondria-dependent apoptosis in TNBC is still not fully understood. Among the miRNAs implicated in apoptotic control, miR-99b-5p has been described as a tumor suppressor in several malignancies, including prostate, gastric, and lung cancers. In these cancers, miR-99b-5p was shown to suppress growth and survival pathways such as PI3K/AKT/mTOR and subsequently promote apoptosis (11–13). Despite its well-documented pro-apoptotic role in other cancer types, the involvement of miR-99b-5p in apoptotic signaling networks in TNBC has not been clearly defined. In this study, we investigate whether miR-99b-5p regulates TRAIL-mediated apoptotic signaling in TNBC by targeting TNFRSF10B (DR5) and associated apoptotic mediators.

## Materials and methods

### Cell lines

For this study, three triple-negative breast cancer (TNBC) cell lines were utilized: MDA-MB-231 (HTB-26), MDA-MB-468 (HTB-132), and BT-20 (HTB-19), all purchased from the American Type Culture Collection (ATCC). MDA-MB-231 cells were cultured in Dulbecco’s Modified Eagle Medium (DMEM) (Hyclone), while MDA-MB-468 and BT-20 cells were cultured in Eagle’s Minimum Essential Medium (EMEM) (Hyclone). For lentivirus preparation HEK-293 cells were also used. The cells were maintained in DMEM (Hyclone). All media were supplemented with 10% fetal bovine serum (FBS) (Hyclone), 1% penicillin-streptomycin (Lonza), and 1% L-glutamine (Lonza). Cultures were maintained at 37 °C in a humidified atmosphere with 5% CO_2_, and routinely tested for mycoplasma contamination using MycoStrip Mycoplasma Detection Kit (InvivoGen). Cell lines were obtained from ATCC and were authenticated by the supplier. STR profiling was additionally performed to confirm identity.

### Transfection of the cells with miRNA mimics and inhibitors

Cells were transfected with miR-99b-5p mimic or scrambled control at a final concentration of 25 nM, and with miR-99b-5p inhibitor or scrambled control at a final concentration of 100 nM. Lyophilized miRNA reagents (5 nmol; Qiagen: MSY0000689, SI03650318; Dharmacon: IH-300658-05-0005, IN-001005-01-05) were reconstituted in RNase-free water to prepare concentrated stock solutions (25 µM for mimic, 100 µM for inhibitor), which were then diluted to the indicated final transfection concentrations. 1 x 10^5^ cells were seeded, transfected, and incubated for 48 hours before proceeding with the experiments. HiPerfect Transfection Reagent (301705, Qiagen) and DharmaFect 1 Transfection Reagent (T-2001-03, Dharmacon) were used for miRNA mimic and inhibitor transfections, respectively. Transfection efficacy was evaluated by qRT-PCR.

### Lentivirus preparation

GFP tagged anti-miR-99b-5p construct (MZIP99b-5p-PA-1), and corresponding non-target control (NTC) lentiviral construct (MZIP000-PA-1) were purchased from SBI (System Biosciences). To produce lentiviral particles, HEK293 cells were transfected with Lipofectamine 2000 (11668019, Thermo Fisher), pPACKH1 HIV Lentivector Packaging Kit (LV500A-1, SBI) and anti-miR or NTC plasmid according to the manufacturer’s protocol. After 48h of incubation the supernatant was collected and filtered through 0.45 μm-pore-size filter.

Cells were transduced with fresh lentiviral supernatant at 100 µL per well of a 6-well plate, in the presence of 8 µg/mL Polybrene for 24 h (#HY-112735, Medchem). After transduction, cells were washed and allowed to recover in fresh medium. Stable cell lines were selected with 2 µg/mL puromycin for 72h (#PUR333.25, Bioshop). Knock-down of miR-99b-5p was validated via qRT-PCR and cells were expanded for further experiments.

### RNA isolation and quantitative RT–PCR

Total RNA was isolated from transfected cells using Qiazol (Qiagen) after 48 hours of incubation. For miRNA expression, RNA was converted to cDNA using the miScript II RT Kit (Qiagen), and transfection efficiency was assessed by qRT-PCR according to the miScript SYBR Green PCR Kit (Qiagen) protocol. For mRNA analysis, cDNA was synthesized using the Transcriptor First Strand cDNA Synthesis Kit (Roche). qRT-PCR was conducted with FastStart Essential DNA Green Master (Roche), and samples were analyzed on the LightCycler^®^ 480 Real-Time PCR System (Roche). Transfection experiments were performed in triplicates, and results were analyzed using the 2-^ΔCT^ method with RNU6 as the reference gene for miRNA expression and GAPDH for mRNA expression normalization. The list of primers used is provided in **Supplementary Table 1**.

### Argonaute-2 based RNA-immunoprecipitation assay

MDA-MB-231 cells were seeded at a density of 4 × 10^6^ cells and transfected with 25 nM miR-99b-5p mimic or negative control using HiPerFect transfection reagent (Qiagen), according to the manufacturer’s instructions. Eighteen hours post-transfection, cells were harvested and subjected to Argonaute-2 (Ago2) RNA immunoprecipitation.

Immunoprecipitation was performed using the Magna RIP™ RNA-binding protein immunoprecipitation kit (17-700, Sigma-Aldrich). Magnetic beads were pre-coated with either IgG (negative control) or AGO2 antibody (03-110, Sigma-Aldrich). The protein lysate was mixed with the magnetic beads and incubated overnight at 4 °C. Following incubation, RNA isolation was performed according to the manufacturer’s protocol.

To validate the efficiency and specificity of Ago2 immunoprecipitation, Western blot analysis was performed. Immunoprecipitated proteins were detected using a mouse monoclonal anti-Ago2 antibody (03-110, Sigma-Aldrich). The presence of Ago2 protein in Ago2 immunoprecipitates, but not in IgG controls, confirmed successful and specific immunoprecipitation (**Figure 2A**). RNA obtained from Ago2 immunoprecipitates was subsequently used for qRT-PCR or next-generation sequencing analyses.

### RNA sequencing

AGO2-RIP-seq was performed using two independent biological replicates per condition (AGO2-IP from miR-99b-5p–mimic transfected vs. control cells). RNA libraries were prepared using the MGIEasy RNA Library Prep Set according to the manufacturer’s instructions. Briefly, the RNA samples obtained from IP experiments (approximately 200 ng) was converted into double-stranded cDNA, followed by magnetic bead–based purification and size selection. End repair, poly-A tailing, and adaptor ligation were subsequently performed, and adaptor-ligated products were amplified by PCR. Library quality and concentration were assessed prior to sequencing, and all libraries were sequenced on the MGI DNBSEQ-T7 platform.

The first step of the RNA sequencing data analysis involved quality control of raw sequencing reads. Paired-end FASTQ files were assessed using FastQC (v0.11.9) to evaluate read quality. Samples passing quality control were subsequently processed with Cutadapt (v3.5) to remove adaptor sequences and low-quality bases. Cleaned reads were aligned to the GRCh38 reference genome using the splice-aware aligner STAR (v2.7.11b), with Ensembl gene annotations. Given the focus on differential gene expression analysis, STAR was run in two-pass mode. Aligned reads were generated in sorted BAM format, and gene-level read counts were obtained using the quant mode option. Differentially expressed genes (DEGs) were identified using the DESeq2 statistical framework via the PyDESeq2 package (v0.4.11) in Python (v3.12.0). Ensembl gene identifiers were converted to gene symbols using the Sanbomics tool (v0.1.0). The details of AGO2-RIP-seq experimental design, sequencing metrics, and analysis parameters is given in **Supplementary Table S2**.

### Western blot

Protein isolation was performed using both *in vitro* and *in vivo* samples. RIPA buffer (#9806, Cell Signaling Technology), supplemented with 1 mM PMSF, was used for protein extraction. Tumor samples obtained from mice were homogenized and centrifuged at 14000 g for 10 minutes, and the supernatant was collected. Protein concentration was determined using the Bradford Assay. Ten micrograms of protein from the obtained samples were loaded onto SDS-PAGE for electrophoresis. Following wet transfer to membranes, primary antibodies were diluted 1:1000 and incubated overnight at 4 °C. The primary antibodies used were: DR5 (#8074, Cell Signaling Technology), BCL-XL (#2764, Cell Signaling Technology, BAK (#12105, Cell Signaling Technology), and ACTB (643802, Biolegend). Rabbit (7074S, Cell Signaling Technology) or mouse (7076S, Cell Signaling Technology) secondary antibodies, diluted 1:10,000, were applied. Membranes were visualized using a LICOR Odyssey imaging system with the WesternBright Sirius HRP Detection Kit (K-12043-D10, Advansta) and analyzed and quantified using Image J (14).

### Cell proliferation assay

To evaluate the effect of miR-99b-5p on cell proliferation, MDA-MB-231, MDA-MB-468, and BT-20 cells (2 x 10^5^ cells/well) were seeded in 6-well plates and transfected the following day with miR-99b-5p mimic, miR-99b-5p inhibitor, or scrambled control according to the transfection protocol. Proliferation was continuously monitored using the IncuCyte cell imaging system (Sartorius, Germany). Phase contrast images were acquired at 6-hour intervals over 96 hours with 10× magnification, imaging nine fields per well at each time point. The data were normalized, graphed, and cell growth quantified using the Phase Object Count analysis module of the IncuCyte system.

### Apoptosis assay

To assess whether miR-99b-5p induces apoptosis in triple-negative breast cancer (TNBC) cells, 2 x 10^5^ cells were seeded in 6-well plates and transfected with miR-99b-5p mimic or scrambled control 24 hours post-plating. Each experiment was performed in triplicates. Following transfection, cells were harvested, washed twice with PBS, and resuspended in 1X Annexin V binding buffer at a concentration of 1 x 10^6^ cells/ml. Annexin V-FITC (5 µl, 640906, BioLegend) and propidium iodide (PI) (5 µl, 0.5 µg/µl, 421301, BioLegend) were then added to the cell suspension and incubated for 15 minutes at room temperature in the dark. After incubation, 400 µl of Annexin V binding buffer was added, and flow cytometry analysis was performed using a NovoCyte^®^ Flow Cytometer (ACEA Biosciences, USA). A total of 100,000 events were acquired, and apoptotic cells were quantified based on Annexin V-FITC and PI staining.

### Cell cycle analysis

To investigate the effect of miR-99b-5p inhibition on the cell cycle in TNBC cells, 2x10^5^ cells seeded in 6-well plates were transfected with miR-99b-5p inhibitor or scrambled control 24 hours after plating. Following transfection, cells were harvested, washed twice with PBS, and fixed with 70% ethanol. The cells were incubated at 4 °C for 30 minutes, then centrifuged at 2000 rpm, and the supernatant was discarded. The cell pellet was washed twice with PBS. Subsequently, 425 µl of PBS and propidium iodide solution (421301, BioLegend) were added, and cells were incubated at 37 °C for 15 minutes. Flow cytometric analysis was performed using a NovoCyte^®^ Flow Cytometer (ACEA Biosciences, USA), and the cell cycle distribution was analyzed. A total of 100,000 events were acquired, and the data were analyzed to determine the percentage of cells in the G0/G1, S, and G2/M phases. Each experiment was performed in triplicates.

### Dual-luciferase reporter assay

To clone sequences containing the binding sites of *TNFRSF10B* (DR5) targeted by miR-99b-5p, appropriate primers were designed for both the wild-type and mutant regions. Plasmids containing these sequences were cloned into the pmirGLO Dual Luciferase miRNA Target Expression Vector (E1330, Promega). HEK-293 cells, seeded at a density of 4×10^4^ cells per well in a 24-well plate, were co-transfected with either the mutant or wild-type plasmids and miR-99b-5p mimic using Lipofectamine 2000 transfection reagent (Invitrogen, 11668019). Renilla and firefly luciferase activities were measured following the Dual-Luciferase Reporter Assay protocol (E1960, Promega). All assays were performed in triplicates, and the data were normalized to Renilla luciferase activity.

### Mouse xenograft model

The *in vivo* xenograft study was performed using the maximum number of animals permitted under ethical and logistical constraints. Female athymic nude mice (6–8 weeks old, 18–20 g) were obtained from the Ankara University Biotechnology Institute animal facility and housed under pathogen-free conditions with ad libitum access to food and water (≤5 mice per cage). All procedures were approved by the Ankara University Animal Care and Use Committee and conducted in accordance with institutional guidelines.

MDA-MB-231 cells stably expressing anti-miR-99b-5p (LV1) or a non-targeting control (Scr) were implanted into the fourth mammary fat pad (5 × 10^6^ cells/injection). Cells were resuspended in PBS and mixed 1:1 with Matrigel (Corning) immediately prior to injection. Tumor growth was monitored by digital caliper measurements every other day. A limited number of animals were excluded due to predefined humane endpoints and/or failure of tumor establishment, resulting in final group sizes of n=4 (Scr) and n=5 (LV1).

Mice were randomly assigned to experimental groups. Tumor measurements and histological analyses were performed by investigators blinded to the treatment groups to minimize bias. The 11-weeks endpoint was selected based on real-time tumor growth kinetics in this model. While MDA-MB-231 xenografts often reach endpoint earlier, LV1-derived tumors showed progressive shrinkage rather than rapid progression; therefore, extended monitoring was required to capture the full regression phenotype. At study termination, mice were euthanized by CO_2_ inhalation followed by cervical dislocation, and tumors were excised, weighed, and snap-frozen for downstream molecular and histological analyses.

### Data acquisition and bioinformatics analysis

Transcriptome profiling data of Breast Carcinoma (BRCA) and corresponding clinical data were obtained from TCGA database. Potential targets of miR-99b-5p were identified using miRWalk (15). A list of apoptosis-related genes was generated through the NCBI Gene database. The expression of target genes was analyzed using Breast Cancer Gene-Expression Miner v5.2 (16) and TNMplot (17), while miR-99b-5p expression levels were assessed using XenaBrowser (18). Survival analysis related to miR-99b-5p was conducted and visualized using StarBase-ENCORI (19). Functional enrichment analysis was performed using WebGestalt based on KEGG pathway annotations (20).

### Statistical analysis

Unless otherwise stated, all *in vitro* experiments were performed with at least three independent biological replicates. Data are presented as mean ± SD. Statistical analyses were performed using GraphPad Prism (version 8.0). Statistical significance was assessed using student’s t-test and two-way ANOVA. p values less than 0.05 were considered statistically significant. The details of statistical tests used for each figure are provided in **Supplementary Table S3**.

## Results

### Clinical relevance and oncogenic role of miR-99b-5p in TNBC

**Figure 1:**
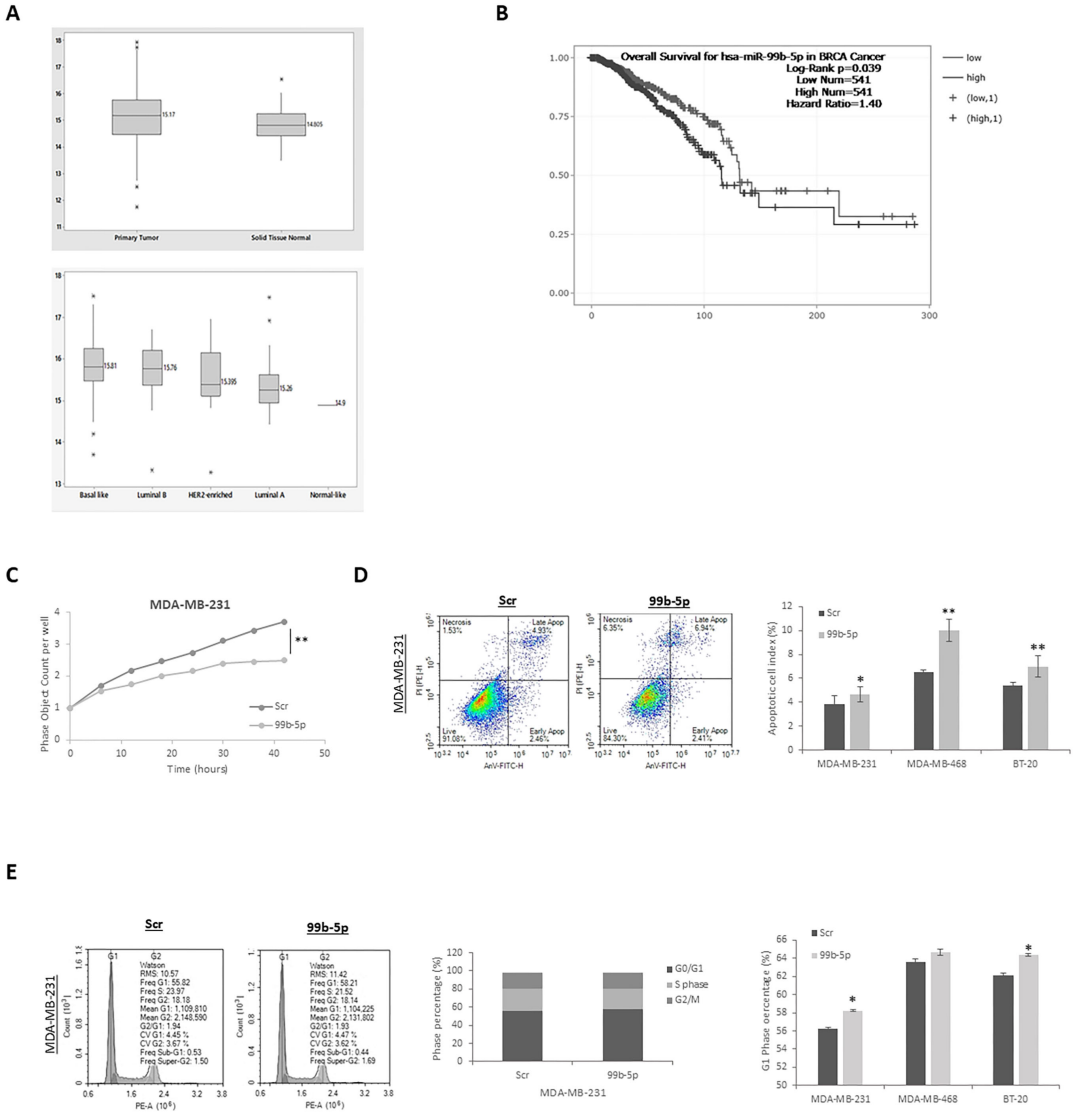
miR-99b-5p inhibition responses of TNBC cell lines. **(A)** Graph showing the expression profile of miR-99b-5p in breast tumors and normal samples. TCGA data analysis showed that miR-99b-5p expression was significantly increased in breast tumors compared to normal samples (t-test, p<0.003) and it is expression is significantly higher in basal like tumors compared to other subtypes (ANOVA, p<0.05) **(B)** Kaplan–Meier overall survival analysis of TCGA patients stratified by miR-99b-5p expression showed that high miR-99b-5p expression is associated with poorer overall survival compared with low-expression tumors. Patients were stratified into high and low miR-99b-5p expression groups (n=541) using the median cutoff; p-values were calculated by log-rank test and HR (95% CI) was obtained from a Cox proportional hazards model (log-rank test, p=0.039). **(C)** Real-time viability measurements showed that downregulation of miR-99b-5p in TNBCs effectively reduced cell proliferation. **(D)** Representative images of apoptotic cells in scrambled control (Scr) and miR-99b-5p inhibitor–transfected TNBC cells are shown. Apoptotic rates were detected by Annexin V-FITC/PI assay and was analyzed using ACEA Novocyte flow cytometer (ACEA Biosciences Inc). Data analysis were performed with NovoExpress 1.5 (ACEA Biosciences Inc.). Bar graphs represent the apoptotic cell index. The results are shown as the mean ± SD of 3 independent biological replicates in each (**p<0.001, *p<0.05). **(E)** Inhibition of miR-99b-5p significantly increased G1 arrest in MDA-MB-231 and BT-20 cells, suppressing malignant cell behavior. All images represent mean of two independent experiments (*p<0.01).

To investigate the clinical relevance of miR-99b-5p in breast cancer (BC), we analyzed its expression profile in the TCGA-BRCA cohort using the UCSC Xena Browser. As shown in **Figure 1A**, miR-99b-5p expression was significantly upregulated in BC tissues compared with normal breast tissues and was markedly higher in basal-like tumors than in other molecular subtypes (ANOVA, p<0.05). Survival analysis performed using the starBase database demonstrated that elevated miR-99b-5p expression was significantly associated with poorer overall survival in BC patients (**Figure 1B**, log-rank test, *p* = 0.039). Collectively, these results indicate that miR-99b-5p is aberrantly overexpressed in BC, particularly in the basal-like/TNBC subtype, and is associated with unfavorable prognosis.

To further elucidate the biological role of miR-99b-5p in TNBC progression, loss-of-function experiments were performed in MDA-MB-231, MDA-MB-468, and BT-20 cells. TNBC cell lines were transfected with a miR-99b-5p inhibitor, followed by *in vitro* evaluation of cellular proliferative capacity. As shown in **Figures 1C–E** and **Supplementary Figure 1**, suppression of miR-99b-5p significantly inhibited cell proliferation and cell cycle progression across all three TNBC cell lines and was accompanied by a marked increase in apoptotic responses. These results demonstrate that miR-99b-5p acts as a pro-oncogenic regulator that promotes malignant phenotypes in breast cancer cells.

### miR-99b-5p directly targets DR5 and modulates TRAIL-R signaling in TNBC

**Figure 2:**
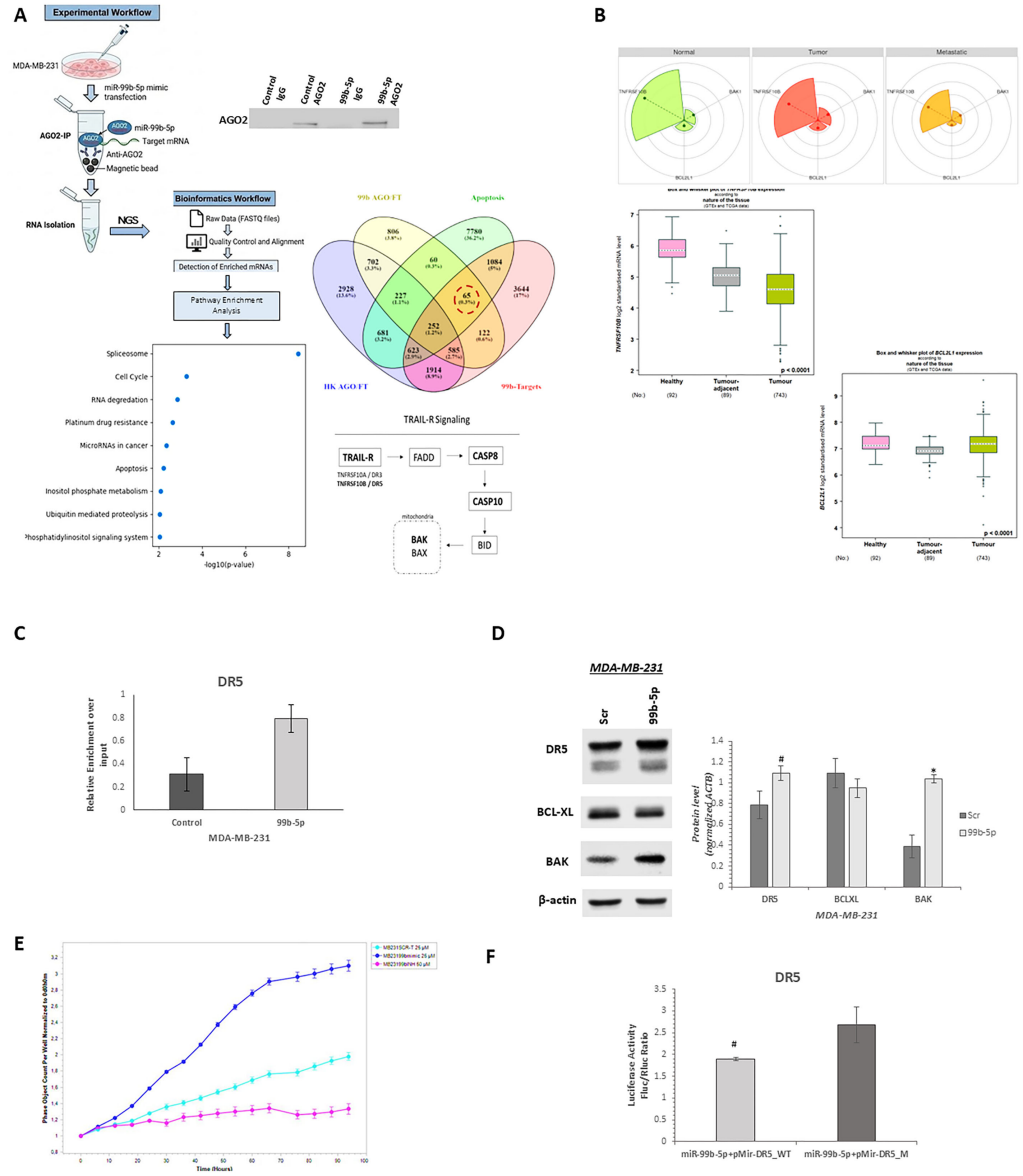
Identification and functional validation of miR-99b-5p-targets involved in TRAIL-R signaling. **(A)** Workflow for identifying candidate miR-99b-5p target genes in MDA-MB-231 cells. RNA immunoprecipitation followed by next-generation sequencing (Ago-RIP-seq) was performed to isolate Ago-bound transcripts associated with miR-99b-5p. These transcripts were intersected with apoptosis-related genes and putative targets predicted by the miRWalk database, resulting in 65 overlapping candidates. Western blot analysis confirmed efficient and specific Ago2 immunoprecipitation, as Ago2 protein was detected in Ago2 IP samples but not in IgG controls. Pathway enrichment analysis of the 65 candidate target genes revealed significant association with the TRAIL receptor signaling pathway, which is schematically illustrated. **(B)** TNFRSF10B (DR5) and BAK1, key pro-apoptotic components of the TRAIL-R pathway, along with the anti-apoptotic gene BCL2L1 (BCL-XL), were selected for further analysis. Bioinformatic analysis of TNMplot RNA-seq data revealed that TNFRSF10B and BAK1 expression was reduced in tumor and metastatic breast tissues compared with normal tissues, whereas BCL2L1 expression did not differ significantly between normal and tumor samples (upper panel). Consistently, analysis of GTEx and TCGA datasets using Breast Cancer Gene-Expression Miner v5.2 showed decreased TNFRSF10B expression in breast tumors, unchanged BCL2L1 expression between normal and tumor tissues, and increased BCL2L1 expression in tumors relative to adjacent normal samples (lower panel). **(C)** RIP-qRT-PCR analysis demonstrating significant enrichment of TNFRSF10B transcripts in Ago immunoprecipitates from MDA-MB-231 cells transfected with miR-99b-5p mimic compared with control, indicating miR-99b-5p–dependent recruitment of DR5 mRNA to the Ago complex. **(D)** Western blot analysis showing that inhibition of miR-99b-5p in MDA-MB-231 cells resulted in upregulation of DR5 and BAK1 protein levels, whereas BCL-XL expression exhibited a decreasing trend without reaching statistical significance. **(E)** Real-time monitoring of cell proliferation using IncuCyte over 96 hours. miR-99b-5p overexpression enhanced proliferative capacity, while miR-99b-5p inhibition significantly suppressed proliferation (In the graph, navy, blue and magents colors represent miR-99b-5p-mimic, Scr and miR-99b-5p-inhibitor transfected cells respectively). **(F)** Luciferase reporter assays demonstrating direct binding of miR-99b-5p to the 3′ untranslated region of TNFRSF10B, validating DR5 as a direct target. Data are presented as mean ± SD from at least three independent experiments. *p<0.01 and #p<0.05 indicate statistical significance.

To elucidate the molecular mechanism underlying the oncogenic role of miR-99b-5p in TNBC, RNA immunoprecipitation followed by next-generation sequencing (Ago-RIP-seq) was performed in MDA-MB-231 cells to identify miR-99b-5p–associated Ago-bound transcripts. Candidate miR-99b-5p target genes were identified by integrating mRNAs significantly enriched in miR-99b-5p–associated Ago immunoprecipitates compared with control Ago-IP samples, bioinformatically predicted targets from the miRWalk database, and apoptosis-related genes. This integrative analysis yielded 65 overlapping candidate targets (**Figure 2A**). Pathway enrichment analysis revealed that these genes were significantly associated with the TRAIL receptor signaling pathway, which is schematically illustrated in **Figure 2A**.

Among the identified candidates, DR5 and BAK1, the key components of the TRAIL-R signaling pathway, were selected for further analysis, together with the anti-apoptotic gene BCL2L1 (BCL-XL). Bioinformatic analysis using TNMplot RNA-seq data from normal, tumor, and metastatic breast tissue samples demonstrated that *TNFRSF10B* and *BAK1* expression levels were reduced in tumor and metastatic tissues, whereas *BCL2L1* expression showed no significant difference between normal and tumor samples (**Figure 2B**, upper panel). Consistently, analysis of GTEx and TCGA datasets using the Breast Cancer Gene-Expression Miner v5.2 revealed decreased *TNFRSF10B* expression in breast tumors, unchanged *BCL2L1* expression between normal and tumor tissues, and increased *BCL2L1* expression in tumors compared with adjacent normal samples (**Figure 2B**, lower panel).

Experimental validation was subsequently performed using RIP-qRT-PCR, which demonstrated a significant enrichment of *TNFRSF10B* transcripts in Ago immunoprecipitates from cells transfected with a miR-99b-5p mimic compared with control, indicating miR-99b-5p–dependent recruitment of *TNFRSF10B* mRNA to the Ago complex (**Figure 2C**). At the protein level, inhibition of miR-99b-5p in MDA-MB-231 cells resulted in a statistically significant upregulation of DR5 and BAK1, while BCL-XL expression exhibited a decreasing trend that did not reach statistical significance (**Figure 2D**).

To assess the functional consequences of miR-99b-5p–mediated regulation, MDA-MB-231 cells were transfected with scrambled control, miR-99b-5p mimic, or miR-99b-5p inhibitor, and cell proliferation was monitored by time-lapse imaging over 96 hours. Cells overexpressing miR-99b-5p displayed enhanced proliferative capacity compared with control cells, whereas miR-99b-5p inhibition significantly suppressed cell proliferation (**Figure 2E**). Finally, luciferase reporter assays confirmed that miR-99b-5p directly binds to the 3′ untranslated region of *TNFRSF10B*, validating DR5 as a direct target of miR-99b-5p (**Figure 2F**).

Taken together, these results demonstrate that miR-99b-5p directly targets TNFRSF10B (DR5), modulates TRAIL receptor signaling, and promotes proliferative phenotypes in TNBC cells.

### miR-99b-5p inhibition suppresses tumor growth and restores TRAIL signaling in TNBC xenograft model

To generate stable miR-99b-5p–inhibited TNBC cells, MDA-MB-231 cells were transfected with the pmiRZip vector carrying a miR-99b-5p inhibitor (LV1 or LV2) or a scrambled control vector (Scr). Successful establishment of stable cell lines was confirmed by GFP fluorescence microscopy (**Supplementary Figure 2A**). qRT-PCR analysis demonstrated efficient and sustained silencing of miR-99b-5p in LV1 and LV2 cells compared with Scr controls (**Supplementary Figure 2B**). Consistent with transient transfection experiments, *in vitro* proliferation assays using the IncuCyte live-cell imaging system revealed that stable miR-99b-5p–inhibited cells (LV1) exhibited significantly reduced proliferative capacity relative to Scr cells (**Supplementary Figure 2C**).

**Figure 3:**
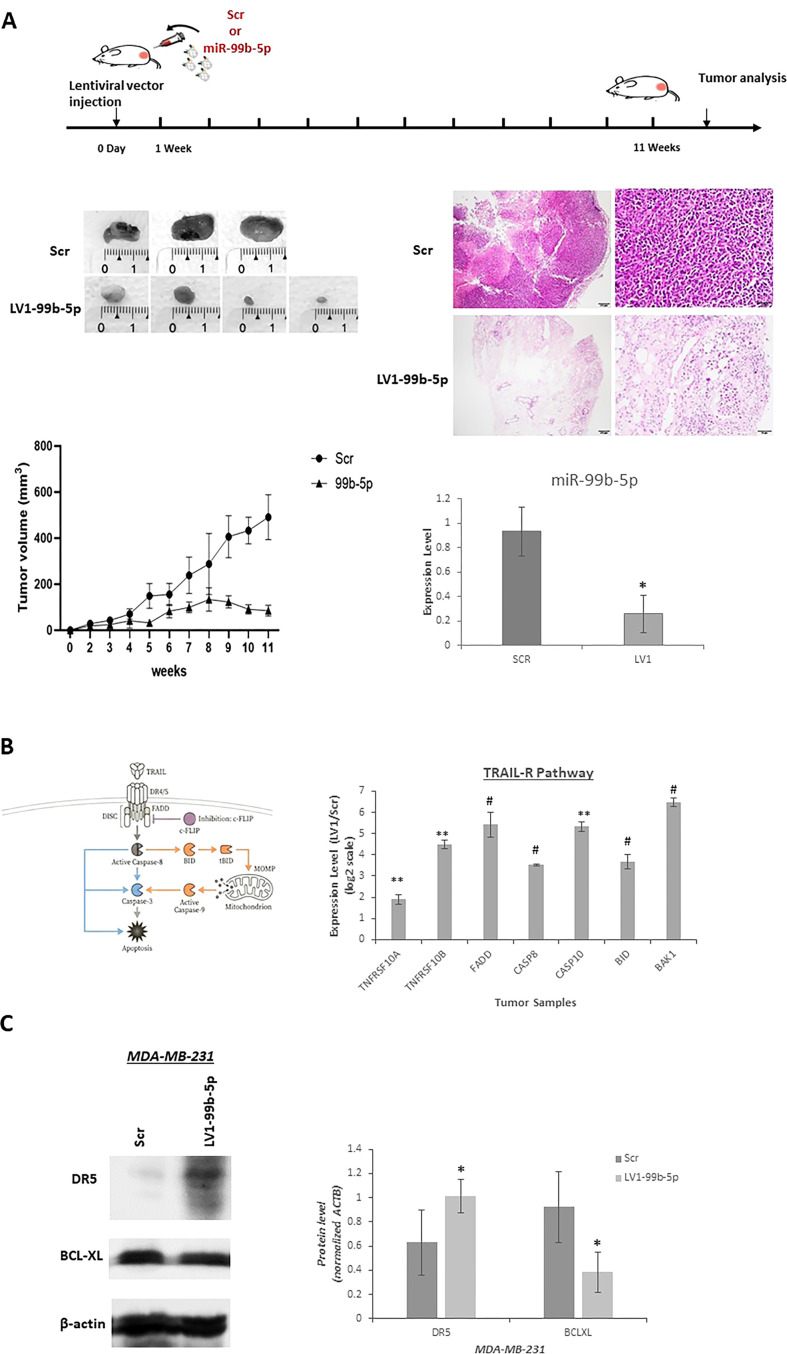
Effect of miR-99b-5p on tumor growth *in vivo*. **(A)** *In vivo* xenograft model of miR-99b-5p inhibition. MDA-MB-231 cells stably expressing a miR-99b-5p inhibitor (LV1) or scrambled control (Scr) were subcutaneously injected into nude mice. Tumor growth was monitored for 11 weeks. LV1-derived xenografts showed significantly reduced tumor growth compared with Scr controls. Representative tumor images and H&E staining demonstrate reduced tumor mass and cellularity in LV1-derived xenografts. The left panel illustrates overall tumor architecture at low magnification (scale bar = 200 µm), whereas the right panel demonstrates cellular morphology at higher magnification (scale bar = 20 µm). qRT-PCR confirmed sustained miR-99b-5p silencing in LV1 tumors. Data are shown as mean ± SD (n = 3); *p < 0.01. **(B)** qRT-PCR analysis of TRAIL receptor signaling–related genes in tumors derived from LV1 or Scr cells. LV1 tumors exhibited increased expression of TNFRSF10A (DR3), TNFRSF10B (DR5), FADD, CASP8, CASP10, BID, and BAK1 compared with Scr tumors, indicating activation of the TRAIL-R signaling pathway (**p<0.0001; #p<0.01.) **(C)** Western blot analysis of tumor lysates showed upregulation of the pro-apoptotic receptor DR5 and downregulation of the anti-apoptotic protein BCL-XL in LV1-derived tumors relative to controls. Data are presented as mean ± SD from at least three independent experiments. *p<0.01 indicates statistical significance.

Following the establishment of stable miR-99b-5p–silenced MDA-MB-231 cells (LV1) and scrambled control cells (Scr), cells were subcutaneously injected into mice, and tumor growth was monitored for 11 weeks. Tumors derived from LV1 cells exhibited significantly reduced growth compared with those formed by Scr cells throughout the observation period (**Figure 3A**). At the experimental endpoint, tumors were excised and measured, revealing that Scr-derived tumors reached significantly larger volumes, with an average diameter of approximately 1,5 cm, whereas tumors formed from LV1 cells were markedly smaller (**Figure 3A**). Histological examination of tumor sections by hematoxylin and eosin (H&E) staining revealed dense and well-defined tumor architecture in Scr-derived xenografts, whereas LV1-derived tumors exhibited reduced tumor cellularity and disrupted tissue organization, consistent with attenuated tumor growth (**Figure 3A**). To confirm sustained miR-99b-5p silencing *in vivo*, miR-99b-5p expression was analyzed in RNA isolated from Scr and LV1 tumor tissues. miR-99b-5p levels were significantly reduced in LV1-derived tumors compared with Scr controls (**Figure 3A**). Furthermore, qRT-PCR analysis of TRAIL receptor signaling related genes demonstrated increased expression of *TNFRSF10A* (DR3), *TNFRSF10B* (DR5), *FADD*, *CASP8*, *CASP10*, *BID*, and *BAK1* in LV1 tumors relative to Scr tumors, indicating activation of the TRAIL-R signaling pathway (**Figure 3B**). At the protein level, analysis of tumor lysates showed that DR5 expression was increased in tumors derived from miR-99b-5p–silenced cells compared with controls, whereas expression of the anti-apoptotic protein BCL-XL was significantly decreased (**Figure 3C**). Overall, these data demonstrate that stable inhibition of miR-99b-5p suppresses tumor growth *in vivo* by restoring TRAIL receptor signaling, thereby attenuating triple-negative breast cancer progression.

## Discussion

TNBC is an aggressive subtype of breast cancer with high rates of tumor recurrence and metastasis. Conventional therapeutic agents available for the treatment of TNBC include doxorubicin, taxanes (paclitaxel, docetaxel), and platinum agents (cisplatin, oxaliplatin) (21, 22). Treatment resistance and lack of specificity are significant limitations associated with these chemotherapeutic agents. The complex cellular mechanisms of cancer cells further hinder the understanding of the molecular puzzle of the disease. Accumulating evidence indicates that miRNAs exhibit distinct expression profiles between malignant and normal tissues and play pivotal roles in modulating cancer-associated phenotypes, functioning either as oncogenic drivers or tumor suppressors depending on cellular context (23). Within this framework, miR-99b-5p has been reported to exert context-dependent functions, acting as either a tumor suppressor or an oncogenic factor associated with enhanced proliferation and unfavorable clinical outcomes. These divergent roles appear to be influenced by tissue specificity, upstream regulatory networks, miRNA isoforms, and the tumor microenvironment. miR-99b-5p has been reported to act as a tumor suppressor in multiple cancer types, inhibiting liver metastasis in colorectal cancer via mTOR downregulation (24), and exerting anti-proliferative effects in gastric cancer by regulating IGF1R (12). Functionally, it can induce autophagy and cell death and enhance therapeutic sensitivity (25). A study identified serum exosomal miR-99b-5p and miR-150-5p as sensitive, specific, and non-invasive biomarkers for the diagnosis of colorectal cancer, with their levels significantly altered in patients and restored postoperatively (26). Interestingly, in the context of breast cancer, emerging evidence suggests that miR-99b-5p may exhibit oncogenic properties, highlighting the tumor type–specific heterogeneity in upstream regulatory mechanisms and target repertoires. The most clinically relevant aspect of miR-99b-5p emerging from our study is its subtype-dependent functional switch. While miR-99b-5p is frequently described as tumor-suppressive in other malignancies, TNBC represents a distinct regulatory context in which survival and apoptotic thresholds are reprogrammed. In this setting, the oncogenic output of miR-99b-5p may reflect (i) differences in upstream transcriptional wiring, (ii) altered availability of competing targets, and (iii) subtype-specific epigenetic states that reshape miRNA–mRNA network topology. Thus, the net phenotype may not dictated by miR-99b-5p alone, but by the TNBC-specific transcriptome that defines which targets are expressed, accessible, and rate-limiting for cell fate decisions.

In breast cancer, large-scale cohort analyses from METABRIC and TCGA have consistently linked elevated intratumoral miR-99b expression with aggressive tumor biology, including enrichment of mTORC1 signaling, activation of cell cycle–related programs (E2F targets, G2/M checkpoint, and mitotic spindle), high histological grade, and genomic instability features such as homologous recombination deficiency and increased mutational burden (27). In our previous study, we demonstrated for the first time experimentally that miR-99b-5p plays a functional role in breast cancer. Clinically, high miR-99b-5p expression was associated with reduced disease-specific and overall survival, particularly in luminal subtypes (28). Moreover, in ER+/HER2+ breast cancer, miR-99b-5p was shown to contribute to endocrine and HER2-targeted therapy resistance by enhancing ER-HER2/EGFR crosstalk and sustaining cell proliferation, whereas its inhibition restored drug sensitivity (28). Consistent with these observations, our present findings demonstrate that miR-99b-5p is significantly upregulated in breast cancer tissues, with particularly pronounced expression in TNBC, and that elevated miR-99b-5p levels are associated with poor patient survival. In luminal tumors, miR-99b-5p may preferentially regulate targets involved in proliferation or hormone-associated pathways, whereas in TNBC, DR5 emerges as a dominant functional target that directly controls the TRAIL apoptotic axis. These results emphasize that miRNA function cannot be generalized across breast cancer subtypes, and that subtype-specific target availability can reprogram the phenotypic output of the same miRNA. Loss-of-function assays further substantiate a pro-tumorigenic role for miR-99b-5p, as its inhibition led to marked suppression of cell proliferation, disruption of cell cycle progression, and induction of apoptotic responses in TNBC cell lines. Collectively, these findings suggest that miR-99b-5p contributes to TNBC aggressiveness by sustaining proliferative signaling and promoting cell cycle progression, highlighting its role as a critical post-transcriptional regulator of TNBC progression.

TNF-related apoptosis-inducing ligand (TRAIL), a member of the TNF superfamily, selectively induces apoptosis in cancer cells while largely sparing normal tissues through engagement of the death receptors TRAIL-R1 (DR4) and TRAIL-R2 (DR5). This tumor-selective apoptotic activity has positioned TRAIL as an attractive anticancer therapeutic candidate. Nevertheless, clinical applications of recombinant TRAIL and TRAIL receptor agonists have been limited by unfavorable pharmacokinetics, short half-life, and intrinsic or acquired resistance mechanisms in cancer cells (29). Although TRAIL-R1 and TRAIL-R2 share substantial sequence homology and activate overlapping apoptotic pathways, their relative contribution to TRAIL-mediated apoptosis appears to be highly context- and cell type–dependent (30–34). Consequently, elucidating the molecular regulators that differentially modulate TRAIL receptor signaling remains critical for improving TRAIL-based therapeutic strategies. MicroRNAs have emerged as key post-transcriptional regulators of apoptotic signaling, including the TRAIL pathway. Previous studies have demonstrated that oncogenic miRNAs can suppress core apoptotic mediators such as DR4/DR5, BID, and APAF1, whereas tumor-suppressive miRNAs frequently target anti-apoptotic factors including MCL1 and XIAP (35). Consistent with this paradigm, increasing evidence indicates that miRNA-mediated regulation constitutes an important layer of control over TRAIL sensitivity across multiple cancer types (36–41). However, the specific contribution of individual miRNAs to TRAIL receptor signaling in TNBC remains incompletely understood. Building on these findings, in our study we revealed the proliferative and anti-apoptotic function of miR-99b-5p through direct regulation of DR5 and modulation of TRAIL receptor signaling in TNBC.

In the present study, integrative RIP-seq and bioinformatic analyses identified TRAIL receptor signaling as a significantly enriched pathway among miR-99b-5p target genes in TNBC cells. Notably, *TNFRSF10B* (DR5) and BAK1, both key pro-apoptotic components of the TRAIL pathway, were identified as direct or functional targets of miR-99b-5p. Publicly available transcriptomic datasets further revealed reduced expression of *TNFRSF10B* and *BAK1* in tumor and metastatic breast cancer samples, supporting the notion that suppression of TRAIL-mediated apoptosis may contribute to tumor progression. In contrast, the anti-apoptotic factor *BCL2L1* (BCL-XL) exhibited either unchanged or increased expression in tumors, consistent with a shift toward apoptosis resistance. Functionally, our *in vitro* data demonstrated that modulation of miR-99b-5p levels exerts a pronounced impact on TNBC cell behavior. While miR-99b-5p overexpression enhanced proliferative capacity, its inhibition suppressed cell proliferation and promoted apoptotic responses. Mechanistically, inhibition of miR-99b-5p resulted in increased DR5 and BAK1 expression at both the mRNA and protein levels, accompanied by downstream activation of apoptotic signaling components, including CASP8 and CASP10. Direct binding of miR-99b-5p to the 3′ untranslated region of *TNFRSF10B* was confirmed by luciferase reporter assays, establishing DR5 as a bona fide target of miR-99b-5p in TNBC cells. Importantly, the tumor-suppressive effects of miR-99b-5p inhibition were recapitulated *in vivo*. Stable silencing of miR-99b-5p in MDA-MB-231 xenografts led to significant tumor growth attenuation and was associated with upregulation of TRAIL receptor pathway components, including increased CASP8 and CASP10 expression, along with a shift toward a pro-apoptotic protein profile characterized by elevated DR5 and reduced BCL-XL levels. These findings indicate that miR-99b-5p functions as a critical negative regulator of TRAIL-mediated apoptosis in TNBC. While an *in vivo* gain-of-function experiment (miR-99b-5p overexpression) would further strengthen causal inference, our results suggest that miR-99b-5p may act as a key modulator linking TRAIL receptor signaling, apoptotic resistance, and tumor growth in TNBC. By directly targeting DR5 and modulating downstream apoptotic machinery, miR-99b-5p appears to facilitate tumor cell survival and proliferation.

Several limitations of our study should be acknowledged. First, our *in vivo* findings were obtained using MDA-MB-231 xenografts, which do not fully recapitulate the complexity of TNBC in patients. Second, while our data demonstrate modulation of TRAIL pathway components (e.g., DR5, BCL-XL, CASP8), we did not directly assess ligand-induced TRAIL signaling *in vivo*; thus, mechanistic conclusions remain cautious. Together, these limitations underscore that our conclusions should be interpreted within the context of the experimental models used and warrant further investigation to more comprehensively define the system-level roles of miR-99b-5p in TNBC. From a translational perspective, our findings raise the possibility that miR-99b-5p inhibition may represent a strategy to restore DR5 expression and lower the apoptotic threshold in TNBC. This suggests that targeting miR-99b-5p could potentially enhance tumor sensitivity to TRAIL/DR5 agonists or other apoptosis-inducing therapies. Given the known limitations of TRAIL-based approaches in solid tumors, often related to insufficient death receptor signaling or adaptive resistance (42, 43), modulation of miR-99b-5p may offer a biologically informed sensitization approach. Moreover, as chemotherapy efficacy partly depends on apoptotic competence, restoring DR5/TRAIL responsiveness could further augment the cytotoxic effects of standard-of-care agents in TNBC. Collectively, these findings expand our understanding of miRNA-mediated regulation of the TRAIL pathway and provide a rationale for future studies exploring miR-99b-5p–targeted strategies in TNBC.

## Data Availability

The datasets presented in this study have been deposited in the Gene Expression Omnibus (GEO) repository under accession number GSE321682 and are publicly available at: https://www.ncbi.nlm.nih.gov/geo/query/acc.cgi?acc=GSE321682.

## Supplementary material

Supplementary Figure 1 Proliferation, Apoptosis and Cell cycle arrest caused by down-regulation of miR-99b-5p in MDA-MB-468 and BT-20 cells. **(A)** MDA-MB-468, and BT-20 cells were transfected with a scrambled control (Scr) or miR-99b-5p inhibitor, and cell proliferation was monitored in real-time using the IncuCyte live-cell imaging system. Scrambled control (Scr) conditions are shown in red lines and miR-99b-5p inhibitor shown by yellow lines. Inhibition of miR-99b-5p significantly reduced proliferation across all TNBC cell lines. **(B)** Apoptotic response in TNBC cell lines (MDA-MB-468 and BT-20) following transfection with a scrmbled control or miR-99b-5p inhibitor, assessed by flow cytometry. miR-99b-5p suppression significantly increased apoptosis. **(C)** Flow cytometry analysis showing that miR-99b-5p suppression induced cell cycle arrest. Data are presented as mean ± SD of three independent experiments.

Supplementary Figure 2 Generation and validation of stable miR-99b-5p–inhibited MDA-MB-231 cell lines. Stable MDA-MB-231 cell lines were generated by transfection with the pmiRZip vector to achieve sustained inhibition of miR-99b-5p (LV1 or LV2) or a corresponding scrambled control vector (Scr). **(A)** Representative GFP fluorescence microscopy images of MDA-MB-231 cells stably transfected with the pmiRZip miR-99b-5p inhibitor vector or control vector, indicating successful establishment of stable cell lines. **(B)** Quantitative analysis of miR-99b-5p expression levels in stable cell lines, demonstrating efficient and sustained silencing of miR-99b-5p compared with Scr control cells. **(C)** Consistent with transient transfection experiments, *in vitro* proliferation of stable MDA-MB-231 cells was monitored using the IncuCyte live-cell imaging system. LV1 cells, stably inhibiting miR-99b-5p, exhibited a significant reduction in proliferation compared with Scr controls. In the graph, Scr cells are represented in green, while LV1 cells are shown in red.

Supplementary Table 1 Primer sequences and amplification details.

Supplementary Table S2 Summary of AGO2-RIP-seq experimental design, sequencing metrics, and analysis parameters.

Supplementary Table S3 Details of statistical analysis.
