# Supplementary figures and images for: miR-99b-5p inhibition drives apoptosis and tumor shrinkage in triple-negative breast cancer: functional characterization through AGO2-RIP-seq and mechanistic insights
Source: Front Oncol. 2026 Apr 22;16:1788447. doi: 10.3389/fonc.2026.1788447 (PMC13143733; doi:10.3389/fonc.2026.1788447)

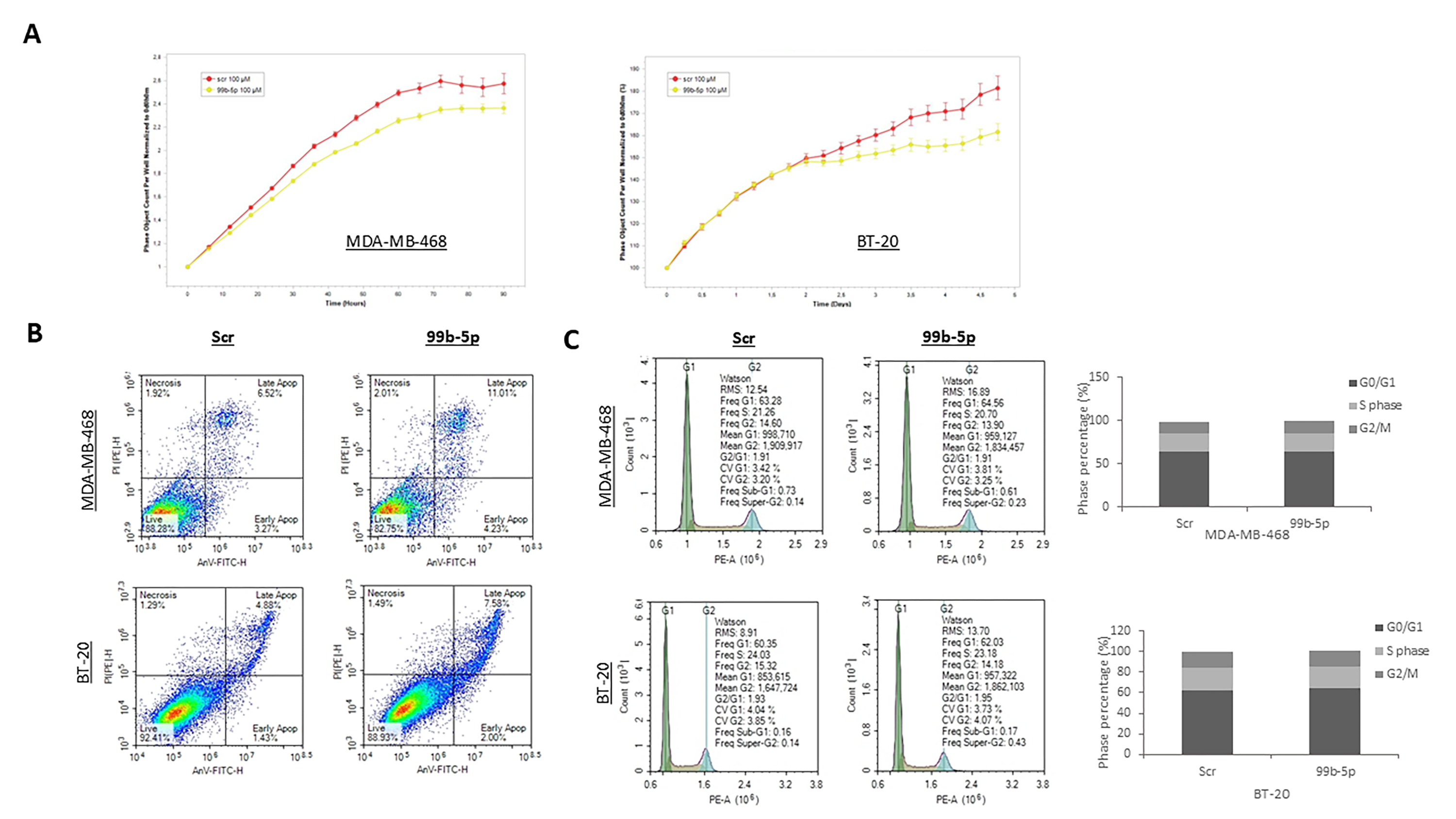

Supplement: Supplementary Figure 1 — Proliferation, Apoptosis and Cell cycle arrest caused by down-regulation of miR-99b-5p in MDA-MB-468 and BT-20 cells. (A) MDA-MB-468, and BT-20 cells were transfected with a scrambled control (Scr) or miR-99b-5p inhibitor, and cell proliferation was monitored in real-time using the IncuCyte live-cell imaging system. Scrambled control (Scr) conditions are shown in red lines and miR-99b-5p inhibitor shown by yellow lines. Inhibition of miR-99b-5p significantly reduced proliferation across all TNBC cell lines. (B) Apoptotic response in TNBC cell lines (MDA-MB-468 and BT-20) following transfection with a scrmbled control or miR-99b-5p inhibitor, assessed by flow cytometry. miR-99b-5p suppression significantly increased apoptosis. (C) Flow cytometry analysis showing that miR-99b-5p suppression induced cell cycle arrest. Data are presented as mean ± SD of three independent experiments. [file Image1.tif]

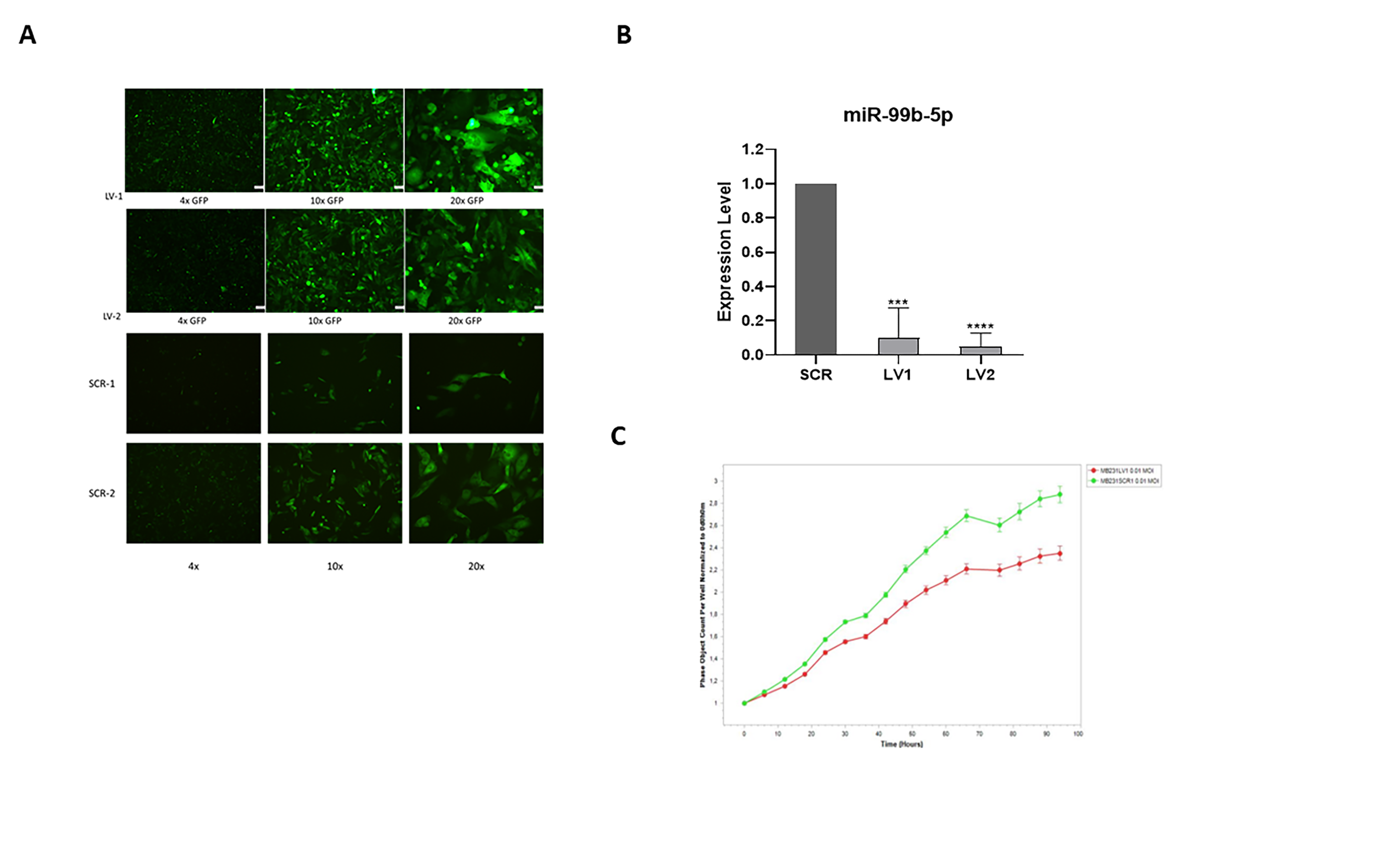

Supplement: Supplementary Figure 2 — Generation and validation of stable miR-99b-5p–inhibited MDA-MB-231 cell lines. Stable MDA-MB-231 cell lines were generated by transfection with the pmiRZip vector to achieve sustained inhibition of miR-99b-5p (LV1 or LV2) or a corresponding scrambled control vector (Scr). (A) Representative GFP fluorescence microscopy images of MDA-MB-231 cells stably transfected with the pmiRZip miR-99b-5p inhibitor vector or control vector, indicating successful establishment of stable cell lines. (B) Quantitative analysis of miR-99b-5p expression levels in stable cell lines, demonstrating efficient and sustained silencing of miR-99b-5p compared with Scr control cells. (C) Consistent with transient transfection experiments, in vitro proliferation of stable MDA-MB-231 cells was monitored using the IncuCyte live-cell imaging system. LV1 cells, stably inhibiting miR-99b-5p, exhibited a significant reduction in proliferation compared with Scr controls. In the graph, Scr cells are represented in green, while LV1 cells are shown in red. [file Image2.tif]
