# Supplementary material for: miR-99b-5p inhibition drives apoptosis and tumor shrinkage in triple-negative breast cancer: functional characterization through AGO2-RIP-seq and mechanistic insights
Source: Front Oncol. 2026 Apr 22;16:1788447. doi: 10.3389/fonc.2026.1788447 (PMC13143733; doi:10.3389/fonc.2026.1788447)
Supplement: Supplementary Table 1 — Primer sequences and amplification details. [file Table1.docx]

**Supplementary Table 1.** **Primer sequences and amplification details.**

| **Gene Name** | **Sequence (5'->3')** | **Tm** | **GC%** | **Product length** | **RefSeq ID** |
| --- | --- | --- | --- | --- | --- |
| MAPK8_F | TAGGCTCAGGAGCTCAAGGA | 59.66 | 55.00 | 113 bp | NM_001323329.2 |
| MAPK8_R | CCGCTTGGCATGAGTCTGA | 60.08 | 57.89 |  |  |
| MAPK9_F | TGCTGCATTTGATACAGTTCTTGG | 60.08 | 41.67 | 105 bp | NM_002752.5 |
| MAPK9_R | AGGACAAGTTCACGATAAGCTCT | 59.49 | 43.48 |  |  |
| MAPK10_F | TTGCCTTTTGTCAGGGATTCG | 59.11 | 47.62 | 137 bp | NM_138982.4 |
| MAPK10_R | GCGCTTGAGAACTGTGAAGG | 59.49 | 55.00 |  |  |
| TNFRSF10A_F | CAAGTTTGTCGTCGTCGGG | 59.15 | 57.89 | 126 bp | NM_003844.4 |
| TNFRSF10A_R | GGTGGACACAACTCTCCCAAA | 60.13 | 52.38 |  |  |
| TNFRSF10B_F | AGACCCTTGTGCTCGTTGTC | 60.25 | 55.00 | 122 bp | NM_003842.5 |
| TNFRSF10B_R | GGGGCTGGACCTCTTTTGTT | 60.18 | 55.00 |  |  |
| CASP8_F | AAGGAGCTGCTCTTCCGAAT | 59.09 | 50.00 | 113 bp | NM_001372051.1 |
| CASP8_R | GCAGAAATTTGAGCCCTGCC | 60.11 | 55.00 |  |  |
| BAK1_F | GAGGAGGTTTTCCGCAGCTA | 59.75 | 55.00 | 108 bp | NM_001188.4 |
| BAK1_R | AGGTTGCAGAGGTAAGGTGAC | 59.65 | 52.38 |  |  |
| BAD_F | GAGTCGCCACAGCTCCTAC | 59.86 | 63.16 | 163 bp | NM_032989.3 |
| BAD_R | GGAGTCCACAAACTCGTCACT | 59.93 | 52.38 |  |  |
| FADD_F | CTGGGGAAGAAGACCTGTGTG | 60.27 | 57.14 | 119 bp | NM_003824.4 |
| FADD_R | GTCCTCGATGCTGTCGATCTT | 59.93 | 52.38 |  |  |
| CASP10_F | TGCAGCACCTCAACTGTACC | 60.25 | 55.00 | 163 bp | NM_032977.4 |
| CASP10_R | TCAGTTTTGGGAAGCGAGTC | 58.12 | 50.00 |  |  |
| BID_F | TCAACAACGGTTCCAGCCTC | 60.53 | 55.00 | 163 bp | NM_001196.4 |
| BID_R | GTCTGCAGCTCATCGTAGCC | 60.87 | 60.00 |  |  |
| GAPDH_F | TTCGACAGTCAGCCGCAT | 60.74 | 57.89 | 171 bp | NM_002046.7 |
| GAPDH_R | TGAAGGGGTCATTGATGGCA | 59.30 | 50.00 |  |  |
